# Functional polymorphism in the 5′-UTR of CR2 is associated with susceptibility to nasopharyngeal carcinoma

**DOI:** 10.3892/or.2013.2421

**Published:** 2013-04-23

**Authors:** QIN FAN, JUN-FANG HE, QI-RUI WANG, HONG-BING CAI, XUE-GANG SUN, XIN-XI ZHOU, HAI-DE QIN, YIN YAO SHUGART, WEI-HUA JIA

**Affiliations:** 1 College of Traditional Chinese Medicine, Southern Medical University, Guangzhou 510515, P.R. China; 2 State Key Laboratory of Oncology in Southern China and Department of Experimental Research, Sun Yat-sen University Cancer Center, Guangzhou 510060, P.R. China; 3 Department of Immunology, Sun Yat-sen University, Guangzhou 510080, P.R. China; 4 Intramural Research Program, National Institute of Mental Health, National Institutes of Health, Bethesda, MD, USA

**Keywords:** nasopharyngeal carcinoma, complement receptor 2, single-nucleotide polymorphism, susceptibility, case-control study

## Abstract

Epstein-Barr virus (EBV)-associated nasopharyngeal carcinoma (NPC) is a squamous cell cancer endemic in Southern China and Southeast Asia. It has been shown that inflammatory and immune responses during EBV infection contribute to the development of NPC. The complement receptor 2 (CR2) gene plays central roles during inflammatory and immune responses and, therefore, is a good candidate susceptibility gene for NPC. We performed PCR-based sequencing to identify multiple single-nucleotide polymorphisms (SNPs) within the exon regions of the CR2 gene in a Cantonese population. Two SNPs were screened in 528 NPC patients and 408 normal individuals to perform a case-control study matched according to age, gender and residence. Furthermore, we cloned the entire 5′-UTR and entire CR2 promoter into a luciferase report system and compared the luciferase activities between the different allelic constructs. A SNP in the 5′-UTR of CR2 (24 T/C, rs3813946) showed a significant association (P<0.01) with NPC in the Cantonese population studied. The subjects were categorized into 2 age groups: group 1, age ≤45 years and group 2, age >45 years. In group 1, the allelic frequencies of 24 T/C in the patients were significantly different from those of the controls (P=0.0034). The odds ratio (OR=1.81) also indicated a higher risk of NPC in individuals who carried the minor allele C. All constructs exerted allelic differences on luciferase activities, but only the susceptible allele +24C construct showed increased activity. Our findings implicate CR2 as a susceptibility gene for NPC and suggest that enhanced CR2 expression may be involved in the oncogenesis and development of NPC.

## Introduction

Nasopharyngeal carcinoma (NPC), a malignancy of epithelial origin, is predominantly endemic in Southern China with an age-standardized incidence rate of 30–50 cases per 100,000 individuals/year (1). The incidence rate for males is as high as >40 cases out of 100,000 individuals/year in the Cantonese-speaking population in Guangdong Province, which is significantly higher than that in the other major dialect-speaking populations who immigrated to Guangdong Province 100 years ago from Northern China (2). Importantly, the incidence of NPC in these populations peaks at the relatively young age of 45 years.

NPC in Southern China is closely linked to infection of the Epstein-Barr virus (EBV) since it was found that titers of antibodies against EBV are elevated in NPC patients, viral DNA and proteins are present in tumor cells with a monoclonal nature, and part of the viral genes or proteins shows transformation potential in lymphocytes and epithelial cell lines (3,4). The mechanism of how EBV enters into the nasopharyngeal epithelium (NE) has not yet been conclusively elucidated; but at least two receptors, the complement receptor type 2 (CR2) (5) and the polymeric immunoglobulin receptor (pIgR) (6), have been proposed. CR2 (CD21), a 145-kDa integral membrane glycoprotein, is the human C3d and EBV receptor. The primary ligand for CR2 is C3d, a processed form of C3 which results from proteolytic cleavage of C3b. CR2 also serves as the EBV receptor on human B cells during EBV infection. It was indicated that a large surface glycoprotein of EBV, gp350/220, is the viral ligand that can bind to CR2 and thus EBV can infect the cells. EBV can also infect recombinant epithelial cells expressing CR2 especially when the cells are in contact with virus-producing lymphocytes (7). In addition, it has been reported that CR is expressed in embryonic NE cells as revealed by RT-PCR, indicating that CR2 may be a sideway of EBV-NE cell infection (8).

Recent studies have shown that CR2 polymorphisms may be associated with a few immunologically mediated diseases such as systemic lupus erythematosus (9,10) and multiple sclerosis (11). Notably, upregulation or downregulation of CR2 was found in different cases. However, to date, no major susceptibility gene such as BRCA1 for breast cancer, has been identified for NPC with significantly increased risk, although several genes including HLA haplotypes (12) and genes of T cell receptors (13), cytochrome P450 2E1 (14), TLR family (15,16), DNA repair enzymes XRCC1 and hOGG1 (17), have been reported to be associated with the risk of NPC. The specific aim of this study was to ascertain whether CR2 is a major NPC susceptibility gene. Our study was conducted using candidate-gene approaches to determine the risk associated with DNA polymorphisms among NPC patients of a Cantonese-based population. Additionally, the basic function of the CR2 promoter and 5′-UTR was also examined in the present study. Our findings indicate that enhanced CR2 expression may be involved in the oncogenesis and development of NPC.

## Materials and methods

*Variation screening.* The CR2 sequence was obtained from the published database of the National Center for Biotechnology Information of USA. All the annotations in the databases for all known exons and untranslated region of CR2 were used. A standard polymerase chain reaction (PCR) method routinely followed in our laboratory, as mentioned later, was employed for all exons, 5′-untranslated region (5′-UTR) and 3′-untranslated region (3′-UTR) of CR2. The primers for the target regions of CR2 were designed with the web-base software Primer 3.0. In the preliminary tests, we amplified and purified the DNA samples from 24 patients with confirmed sporadic NPC in Southern China. These samples included 48 chromosomes, providing at least a 95% confident level to detect alleles with frequencies >5%. The PCR products were then sequenced by ABI^®^3730XL Automatic Sequencer (Applied Biosystems, Foster City, CA, USA). We used the Polyphred/Phredphrap/Consed software package to identified single-nucleotide polymorphism (SNP) candidates that were then confirmed by two independent observers. These SNP positions and individual genotypes were further confirmed using re-amplifying and sequencing and again reversely.

*Study population.* The subjects of this case-control study consisted of 528 patients with histopathologically confirmed NPC and 408 population controls. All subjects were unrelated ethnic Cantonese from the Cantonese-speaking population in Nanhai, Foshan, Shunde, Qingyuan, Sihui and Luoding regions of Guangdong Province, China. Patients were recruited consecutively from December 2003 to October 2004 at the Sun Yat-sen University Cancer Center, Guangzhou, China. The average age for all 528 patients at diagnosis of NPC was <50 years. Population controls were cancer-free individuals and non-relatives of the patients; and they were randomly selected with a community cancer-screening program for early detection of cancer during the same period as the cases were enrolled. The selection criteria for control subjects included: i) no individual history of cancer; ii) frequency matched to NPC cases according to gender, age (±5 years); iii) the residential region; and iv) the time period for blood sample collection. At recruitment, informed consent was obtained from each subject, and each participant was then interviewed to collect detailed information on demographic characteristics. This study was approved by the Human Ethics Approval Committee of the Cancer Center of Sun Yat-sen University, China.

*Genotype analysis.* Variations among genotypes were determined by the PCR-based DNA direct sequencing. Genomic DNA was extracted from the peripheral blood samples of all case and control subjects using the DNAzol kit according to the manufacturer’s protocol (Gibco-BRL, Life Technologies, Carlsbad, CA, USA). The genomic DNA samples were amplified by PCR using a GeneAmp^®^ 9700 PCR system (Applied Biosystems). The 20-μl PCR reaction mixture contained 20 ng DNA, 0.2 μM of each primer, 200 μM of each deoxynucleotide triphosphate, 1.5 mM of MgCl_2_, 0.3 units of Taq DNA polymerase with 1X buffer (Qiagen, Chatsworth, CA, USA). The primers used to amplify DNA containing the 5′-UTR region were 5′-agtgggttgcgtggtcaaaa-3′ and 5′-agtggggacaatcagga cca-3′, which produce a 550-bp fragment; the primers used to amplify DNA containing exon 10 were 5′-ataccgtccaggaaac aac-3′ and 5′-cctctttccatgatgcagtt-3′, which produce a 606-bp fragment; the primers used to amplify DNA containing exon 17 were 5′-ggtggactggatcaaatcag-3′ and 5′-gggcttccttttgtatag cac-3′, which produce a 420-bp fragment. The reactions were carried out under the following conditions: an initial melting step of 15 min at 95°C; followed by 30 cycles at which each one consisted of 30 sec at 94°C, 30 sec at 60°C and 45 sec at 72°C, respectively; and a final elongation step of 5 min at 72°C. Automatic DNA sequencing was performed on an ABI^®^ 3730XL Automatic Sequencer (Applied Biosystems) using direct PCR products of the samples according to the manufacturer’s protocol. The raw data were analyzed using Sequencing Analysis software V3.3 on a MAC operating system v9.1, Phred-Phrap-Consed software package, DNAStar/TaqMan software package and Chromas. All genotyping was performed blinded to the case/control status, and blinded quality control samples were inserted to validate the genotype. Random samples (10%) of cases and controls were sequenced twice by different investigators to corroborate the findings, and the reproducibility was 100%.

*Preparation of the reporter constructs.* The entire 1327 bp (−1252 to +75) of the CR2 promoter was amplified using the following primers: PPF, 5′-gatggtaccATGAAGCTTCCAG CCAAAG-3′ and PPR, 5′-gatctcgagCGGCGGGATGCGTT CCGAGA-3′. The sequence (−57 to +94, 151 bp) containing 5′-UTR of CR2 was amplified using the primers PUF, 5′-gat ggtaccTGCGCTCAGAACTAGCACGTGT-3′ and PUR, 5′-gat ctcgagGCCACGGCCGAAGCCCCCGCG-3′. The design of the primers incorporating the *Kpn*I and *Xho*I sites at the 5′ end (underlined primers) enabled cloning into the firefly luciferase expression plasmid (pGL3-basic; Promega, Madison, WI, USA). Distinct DNA from blood samples, each containing a different genotype of 24T and C, were used as templates to obtain the promoter and 5′-UTR sequence for cloning. The PCR conditions consisted of 0.2 μM of each primer, 200 μM of each deoxynucleotide triphosphate, 1.5 mM of MgCl_2_, 0.3 units of Taq DNA polymerase with 1X buffer (Qiagen) in a final volume of 20 μl. PCR was performed at 94°C for 2 min, followed by 25 cycles at 94°C for 30 sec, 55°C for 1 min and 72°C for 1 min. The final extension step was at 72°C for 5 min. The pure PCR products were digested with *Kpn*I/*Xho*I and then were cloned into the *Kpn*I/*Xho*I site of pGL3-basic plasmids. We discriminated them by sequencing, and named them PWILD and PMUT for the variants of the promoter constructs, and UWILD and UMUT for the variants of the 5′-UTR construct. Recombinant DNA cloning was performed according to standard methodologies (18). Plasmid DNA for the transfections was prepared using the Qiagen Plasmid Maxi kit (Qiagen, Hilden, Germany) according to the manufacturer’s instructions. All luciferase constructs were verified by sequencing.

*Cell-based gene expression and luciferase assay.* Human NPC C666 cells were grown in RPMI-1640 medium (Gibco Laboratories, Grand Island, NY, USA) containing 10% heat-inactivated fetal bovine serum (FBS) and 1% L-glutamine (Sigma, Chemical Co., St. Louis, MO, USA) in a humidified chamber maintained at 37°C containing 5% CO_2_. Cells in a 24-well plate were grown to 70% confluence prior to the transfections. Each construct (1.5 μg) was cotransfected with pRL-EF (25 ng) as an internal control using 2 μl of Lipofectamine 2000 (Invitrogen, Carlsbad, CA, USA). The control plasmid contained the pGL3-control, rather than the construct. This control was cotransformed with the pRL-EF plasmid as a positive control for luciferase expression. Forty-eight and sixty hours later, the cells were harvested and assayed for firefly luciferase and *Renilla* luciferase activities using Dual Luciferase Reporter Assay system (Promega). The relative firefly luciferase activities were normalized against the *Renilla* luciferase activities.

*Statistical analysis.* For each polymorphism, deviation of the genotype frequencies in the control subjects from those expected under Hardy-Weinberg equilibrium was assessed using the standard Chi-square test. The distributions of genotypes between case patients and control subjects were compared using the Monte Carlo approach (19) (data not shown). We estimated the cancer risk associated with the alleles, genotypes as odds ratios (ORs) and 95% confidence intervals (CIs) with adjustment particularly for age using SPSS software (version 11.0). All statistical tests were two-sided, and the probability level <0.05 was used as the criterion of significant statistical difference. In *in vitro* experiments, the transfection and luciferase assays were performed in triplicate. The data were analyzed using the ANOVA test. Statistical significant differences were considered for P<0.05, and data points represent the means ± SD.

## Results

*Study sample and variation screening.* The blood DNA samples from 528 sporadic NPC cases and from 408 controls were screened for possible SNPs of CR2. No significant statistical differences in the age (P=0.0795) and gender (P=0.6266) distributions were observed between the NPC patients and controls, suggesting that the frequency matching was adequate (Table I). In order to identify SNPs with a >5% frequency in the NPC cases, we used a relatively small sample containing 24 NPC cases. We sequenced all the coding exons, the promoter region and the exon-intron boundary regions of CR2 for detection of possible alterations. We detected 3 SNPs in CR2 including 1 in exon 1 (24 T>C, rs3813946), 1 in exon 10 (18650 G/A, rs1048971) and 1 in IVS17 (25775 T/A, rs17258996), all of the SNPs have already been registered in the NCBI database. Since 24 T>C in 5′-UTR and 25775 T/A in IVS17 were fully linked as a haplotype of all the study population, and therefore had the same frequency totally different from that in NCBI, only 24 T>C was assessed in the following analysis.

*Association of individual SNPs with the risk of NPC.* We focused on 2 SNPs and detected their frequency in all of the subjects including the 528 NPC cases and 408 controls. When comparing the allelic frequency between the cases and controls, SNP 24 T>C was found to have a significant difference among the cases and controls (P=0.0221, Table II). After categorizing the cases into 2 age groups of ≤45 and >45 years, the minor allele C frequency was 12.4% of cases in the ≤45-year age group, whereas it was 6.9% in the controls. The allelic frequencies in the cases were significantly different from those of the controls (P=0.0034, Table III). The odds ratio (OR=1.81) also showed a higher risk of NPC in individuals carrying the minor alleles. The distributions of the other SNP 18650 G/A was not different between the cases and controls (Tables II and III).

**Figure 1:**
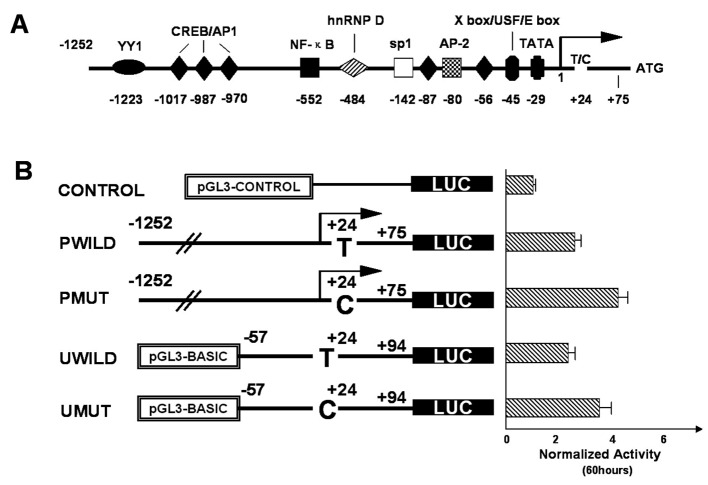
Transcriptional effects of SNP 24T/C by expression of reporter luciferase gene activity. (A) Transcription factor binding sites within the proximal CR2 promoter critical for regulation of basal transcription (32). Shown are nucleotide positions and the functional role of localized elements, TATA box, transcriptional initiation site and position of SNP 24T/C. (B) Transcriptional activity of constructs expressing the major or minor 24T/C SNP allele. Results shown represent mean promoter activity ± SE and are expressed as normalized transcriptional activity of recombinant constructs with P group and U group 60 h after transfection. Expression of the minor +24 C allele resulted in a marked increase in transcriptional activity compared with expression of the major T allele in the two groups (P<0.05). The experiments were repeated 3 times (n=3).

*Comparison of luciferase activity among the constructs.* CR2 5′-UTR variants used in the present study were obtained by PCR and were cloned into the pGL3/LUC basic or promoter vectors (Fig. 1) as described in Materials and methods. To compare 5′-UTR-mediated gene expression by each of the four constructs, the C666 cells were transiently transfected with the recombinant luciferase vectors (PMUT or PWILD, UMUT or UWILD constructs, pGL3-control and pEF-RL). The *Renilla*-normalized luciferase activities of the P group (PMUT and PWILD) and U group (UMUT and UWILD) constructs were as follows: 48 h after transfection, the reporter gene activity of the MUT construct was higher than that of the WILD construct (P<0.05) either in the P or U group. Moreover, 60 h after transfection, the normalized luciferase activity was markedly different, with the PMUT/UMUT construct increasing the reporter gene activity by ~40–50% compared to that of the PWILD/UWILD construct (P<0.01; Fig. 1).

## Discussion

Genetic background plays an important role in the development of NPC, which is an endemic multifactorial genetic disease resulting from gene-environment-EBV interaction. Genetic susceptibility plays a critical role in determining the individual risk of NPC (20). We are in the process of systematically searching for genetic susceptibility genes for NPC. Besides those genes involved in carcinogen metabolism, such as CYP2E1, genes regulating immune response against microbial infection are also our priority (14).

EBV is a ubiquitous herpes virus that infects more than 90% of the human population and establishes a life-long viral persistence in the host. EBV has been consistently identified as an important risk factor for NPC, with a dose-response relationship between EBV antibodies and NPC risk. The single clonally derived viral genome can be found in all endemic NPC cells (21). Altered cell signaling is the molecular basis for EBV infection-induced aberrant cell proliferation. The EBV DNA may persist for its lifetime in an episomal form in the host carrier cells. The virus-encoded protein products or the virus-transcribed non-coding regulatory dsRNAs can activate the transcription of otherwise silenced cellular genes, which leads to the synthesis of enzymes capable of promoting viral and cellular DNA replication. Thus, these proteins or/and regulatory dsRNAs block apoptosis and drive host cells toward division and immortalization. Particularly at later stages of oncogenesis, the viral-encoded proteins and the viral-transcribed non-coding regulatory dsRNAs, inducing false signaling and activation of the proliferation pathways, bring the previously infected cells into a full transformation burst (22).

CR2 may be a good candidate susceptibility genes for NPC due to its function of regulating the immune response and mediating EBV infection. Chronic EBV infection that may lead to tumorigenesis in nasopharyngeal epithelium cells is mediated through recognition of EBV stimuli by CR2 and pIgR receptors. Improper regulation or compromised function of CR2 may contribute to NPC. Our study represents the first comprehensive evaluation of an association between sequence variants in the 5′-UTR of CR2 and NPC. Variations in gene 5′-UTR can lead to altered gene expression levels, and potential disequilibrium of the normal cellular machinery. In the present study, we identified DNA variants in the 5′-UTR region of CR2 that may account for variability of CR2 transcription regulation among individuals with NPC, namely genetic variations due to mutations in the CR2 5′-UTR. The variant 24C conferred a nearly 2-fold increase in the risk for developing NPC (OR=1.81; 95% CI, 1.21–2.70) in the investigated population. Although the observed 2-fold increase in risk is modest, our finding is intriguing as genes in multiple pathways alter the risk for NPC, and each individual gene likely contributes only a modest risk. This phenomenon is also observed in other complex diseases (23). It is interesting that the risk associated with the mutation 24C genotype was more pronounced in younger (45 years or younger) individuals. These results suggest that the 24C substitution in the 5′-UTR of exon 1 in CR2 may act as a genetic susceptibility factor for NPC.

Initiation of translation is one of the most important steps that may influence the level of gene expression, and 5′-UTR sequences may greatly contribute to this step. In fact, recent studies have shown that 5′-UTR plays an important role in the regulation of gene expression (24) in a variety of organisms (microbes, plants and animals). The 5′-UTR-mediated regulation has been suggested to modulate gene expression through both stimulatory (25) and inhibitory mechanisms (26), including influencing RNA transcription (27), post-transcriptional modification of RNA (secondary structure and mRNA stability) (28) and alteration of translational efficiency (29). Indeed, a recent study demonstrated that 5′-UTR plays an important role in post-transcriptional modification and/or translation. The 5′-UTR has also been shown to affect translation efficiency through a cap-independent internal ribosome entry site (IRES)-mediated mechanism (30). Although this novel mechanism of the translation initiation was first discovered in picornaviral RNAs, increasing evidence indicates that certain eukaryotic mRNAs also apply a similar mechanism. The IRES-mediated translation initiation requires specific sequences that can form a Y-shaped secondary structure and a short stem loop near the start of the AUG codon (31). Approximately 6.4% of human 5′-UTR sequences contain IRES and these leader sequences are usually >200 bp in length. However, it is currently unknown whether shorter leader sequences (with <200 bp), such as the hSP-A 5′-UTR variants under study, are involved in IRES-mediated translational mechanism.

It has been shown that the activity of the CR2 promoter is induced in IM-9 B cells through the protein kinase A- and protein kinase C-signaling pathways, and by anti-CD40 Ab and IL-4 (32). The transcription factors that were subsequently identified to play a role in this induced expression include AP-1, CREB and an X box/E box-binding protein. In addition, the protein kinase A and protein kinase C-responsive heterogeneous nuclear ribonucleoprotein D (hnRNP D)^3^ was found to specifically bind to a novel element in the CR2 promoter. It was reported that truncation of promoter sequences from −1252 to −57 did not have a significant effect on promoter activity. Thus, the −57/+75 construct displayed activity similar to that of the −1252/+75 full promoter. These findings clearly suggest that critical positive promoter elements are present in the −57/+75 region, which may contain potential regulatory elements such as CREB/AP-1 half-site, X box and E box. However, these findings do not exclude the possibility that promoter elements upstream of −57 play a role in regulating CR2 promoter activity.

Since the SNP 24T>C is located in the −57/+75 region of 5′-UTR, we investigated its significance on gene expression in the present study. The results of transient transfection indicated that the 5′-UTR of CR2 differentially influences the expression of the reporter gene in C666 cells. The activity of PMUT and UMUT was significantly increased compared with the WILD constructs, indicating that the sequences within the CR2 5′-UTR, and specifically those surrounding the 24 position, regulate CR2 promoter activity and the variation in this region may lead to increases in CR2 expression. The higher levels of CR2 may be a risk of NPC susceptibility, since upregulated CR2 expression may enhance the EBV infection through either B cells or epithelial cells thus mediating the development and oncogenesis of NPC. Nevertheless, we do not exclude the possibility that other mechanisms may also contribute to the increase in CR2 levels during NPC onset.

The SNP 24T>C was also reported as a susceptible factor of systemic lupus erythematosus (9,10). By constructing −315/+75 promoter region into the luciferase report system and the transfection constructs into the Raji B lymphoblastoid cell line, expression of the major 24 T allele resulted in a 2-fold increase in transcriptional activity compared with expression of the minor C allele. The differences between the above study and ours may be due to the result of multiple factors, including alterations in different cell lines and different cloned regions of the promoter.

## Figures and Tables

**Table I:** Distribution of characteristics of the study subjects.

	Cases (n=528)	Controls (n=408)	
Characteristics	n (%)	n (%)	P-value^a^
Gender			
Male	388 (73.48)	294 (72.05)	0.6266
Female	140 (26.51)	114 (27.94)	
Age (years)			
≤45	375 (71.02)	267 (65.44)	0.0795
>45	153 (28.98)	141 (34.56)	
Mean age	42±18	43±10	

a P-values obtained from Chi-square tests.

**Table II:** CR2 genotype frequencies of selected SNPs and their contributions to the risk of NPC.

	Cases (n=528)	Controls (n=408)		
Genotype	n (%)	n (%)	P-value	OR (95% CI)
24 T/C				
TT	415 (78.6)	343 (84.1)		
TC	101 (19.1)	60 (14.7)	C−/C+^a^	
CC	12 (2.3)	5 (1.2)	0.0345	1.44 (1.03–2.01)
Allele C frequency	0.118	0.086	0.0221	1.43 (1.05–1.95)
18650 G/A				
GG	376 (71.2)	309 (75.7)		
GA	117 (22.2)	72 (17.6)	A−/A+^b^	
AA	35 (6.6)	27 (6.7)	0.1214	1.26 (0.94–1.69)
Allele A frequency	0.177	0.154	0.1924	1.18 (0.92–1.51)

a C−/C+ means TT genotype vs. others;

b A−/A+ means GG genotype vs. others.

**Table III:** CR2 genotype frequencies of selected SNPs stratified by age and their associations with risks of NPC.

	Cases (n=375)	Controls (n=267)		
Genotype	n (%)	n (%)	P-value	OR (95% CI)
24 T/C				
Age ≤45 years				
TT	294 (78.4)	233 (87.3)		
TC	73 (19.5)	31 (11.6)	C−/C+^a^	
CC	8 (2.1)	3 (1.1)	0.0039	
Allele C frequency	0.124	0.069	0.0034	1.81 (1.21–2.70)
18650 G/A				
Age >45 years				
GG	124 (81.0)	118 (83.7)		
GA	24 (15.7)	16 (11.3)	A−/A+^b^	
AA	5 (3.3)	7 (5.0)	0.5531	
Allele A frequency	0.111	0.106	0.8541	1.05 (0.62–1.77)

a C−/C+ means TT genotype vs. others;

b A−/A+ means GG genotype vs. others.
